# Dynamics of Opinion Forming in Structurally Balanced Social Networks

**DOI:** 10.1371/journal.pone.0038135

**Published:** 2012-06-19

**Authors:** Claudio Altafini

**Affiliations:** SISSA, International School for Advanced Studies, Trieste, Italy; University of Namur, Belgium

## Abstract

A structurally balanced social network is a social community that splits into two antagonistic factions (typical example being a two-party political system). The process of opinion forming on such a community is most often highly predictable, with polarized opinions reflecting the bipartition of the network. The aim of this paper is to suggest a class of dynamical systems, called monotone systems, as natural models for the dynamics of opinion forming on structurally balanced social networks. The high predictability of the outcome of a decision process is explained in terms of the order-preserving character of the solutions of this class of dynamical systems. If we represent a social network as a signed graph in which individuals are the nodes and the signs of the edges represent friendly or hostile relationships, then the property of structural balance corresponds to the social community being splittable into two antagonistic factions, each containing only friends.

## Introduction

In social network theory, a community of individuals characterized by friendly/hostile relationships is usually modeled as a signed graph having the individuals as nodes and their pairwise relationships as edges: an edge of positive weight expresses friendship, one of negative weight aversion or hostility [1], [2]. According to Heider theory of structural balance [3], in a balanced community the role of friends and enemies, determined locally by the bipartite relationships, is perfectly defined also on triads, and is equivalent to all length-3 cycles having positive sign. Since the sign of a cycle is the product of the signs of its edges, positive sign corresponds to an even number of negative edges along a cycle. Heider’s original definition for triads can be generalized to larger groups of individuals using the graph-theoretical formulation of Cartwright and Harary [4]: the lack of structural tensions corresponds to all cycles of the signed graph being positive. Also for this more general definition (the one adopted in this paper) structural balance implies a lack of ambiguity in the way each individual classifies each other individual as a friend or as an enemy. An equivalent characterization is in fact that the network splits into two factions such that each faction contains only friendly relationships while individuals belonging to different factions are linked only by antagonistic relationships [1].

Following [4], in a perfectly balanced community it is reasonable to assume that for a person the point of view of a friend influences positively the process of forming an opinion about a subject; the opposite for an adversary. Quoting [4]: “the signed graph depicting the liking relations among a group of people will, then, also depict the potential influence structure of the group”. Under this hypothesis, it is plausible to deduce that the outcome of an opinion forming process overlaps with the bipartition of the network: opinions are homogeneous within a faction and opposite with respect to those of the other faction. In this paper we ask ourselves what kind of dynamics is suitable to represent this process of forming an opinion in a structurally balanced world of friends and adversaries and what are the dynamical properties that render the process so highly predictable in presence of structural balance.

In terms of dynamical systems, we can think of “influence” in the sense mentioned above as a directional derivative in opinion space, and of the Jacobian matrix of partial derivatives as the collection of all these influences. The principle stated above that the influences among the members of the community are depicted by their social relationships corresponds to identifying the signs of the entries of the Jacobian with those of the “sociomatrix” i.e., of the adjacency matrix of the signed graph describing the social network. The role played by friends and adversaries is assumed to be free from ambiguities, and this corresponds to constant sign of the partial derivatives in the entire opinion space. In dynamical systems theory, the systems whose Jacobians are sign constant at all points and such that the associated signed graph consists only of positive cycles form an important class of systems, called monotone systems [5]–[7]. Monotone systems are well-known for their dynamical properties: they respond in a predictable fashion to perturbations, as their solutions are “ordered” in the sense that they do not admit neither stable periodic orbits nor chaotic behavior [7]. Owing to their order-preserving flows, in many aspects monotone systems behave like 1-dimensional systems. Such notions of order are very appropriate for structurally balanced social networks, for which the pattern of opinions is completely predictable from the signed graphs depicting the social relationships [4]. Scope of this paper is to make the link between structural balance theory and monotone dynamical systems theory clear and formally precise.

A classical example where structural balance theory applies is two-party (or two-coalition) political systems [8], [9]. In these systems we too often see that opinions within a faction are monolithic and antipodal to those of the other faction, and that discussion among the two factions is a wall-against-wall fight. Other cases in which structural balance has been suggested to correctly reproduce the phenomenology of a social community are for example the pattern of international alliances leading to World War I [10] (one could easily add the armed peace of the post-war Iron curtain [11]), duopolistic markets, rival business cartels [12], various case studies from anthropology [13] and social psychology [14]–[16]. See [1], [2] for a more complete list of examples. In other contexts, notably in biological networks [17]–[20] and in on-line social networks [21]–[23], structural balance is not exact. One can then try to quantify this amount of unbalance [17], [19], [21], [24], [25], or study dynamical evolutions of the edge signs that lead to structural balance [10], [26], [27]. These types of dynamics are fundamentally different from those investigated here, as our sociomatrices are and remain structurally balanced for all times.

If a major feature of a structurally balanced world is that the members of a community are influenced in their decision by the social network they form, a series of other properties of these systems admit interpretations in terms of monotone dynamics. One such property is that a small germ of opinion seems to be propagating unavoidably to the whole community if the network is connected. In structurally balanced systems, this often seems to be happening only due to the process of decision forming itself, regardless of the intrinsic value of the opinion (think of some decisions in the aforementioned two-party political systems). Monotone systems, thanks to their order-preserving solutions, also exhibit this behavior. We will show how for these systems the individual who seeds an idea first has a strong competitive advantage over both friends and competitors.

The signed graphs used in social network theory can be either undirected or directed [1], [2]. In the present context, an undirected edge corresponds to a mutual relationship (and mutual influence) between the two individuals connected by the edge, while a directed edge corresponds to an influence which is not reciprocated. In many instances of social networks, in fact, not all individuals have the same power of persuasion over their peers. In particular, the fact that an “opinion leader” may be influential for the opinions of his neighbors on the network (both friends and adversaries), does not mean that the implication has to reciprocate. Both the concepts of structural balance and of monotonicity extend to directed graphs in a similar manner. Also the graphical tests available in the literature coincide [1], [7].

If influences are associated with edges of the social network, it means that an individual with zero in-degree is unaffected by the opinion of the community (one with zero out-degree is instead unable to influence the community). At the other extreme, highly connected individuals are those influencing (or being influenced) the most. In particular, strong connectivity of a network means that all individuals have some influence power and are at the same time influenced by the community. A monotone dynamical system on a strongly connected graph is called strongly monotone [5]. The main characteristic of strongly monotone systems is that the order in the solutions is strict. This corresponds to the property that all individuals in a strongly connected structurally balanced graph must necessarily take side: neutral opinions are not possible on such social networks.

Although the strength of the opinions at steady state depends on the precise functional form chosen for the dynamics, we already mentioned that in general the individuals with the highest in-degree achieve the strongest opinions. In our models this is true regardless of whether their relationships are friendly or hostile. We interpret this property by observing that both monotonicity of a system and structural balance of a social network are invariants of a particular class of operations which, for analogy with Ising spin glasses in Statistical Physics [28], we call gauge transformations. Consider the signed graph representing the social network and a cut set that splits the graph into two disconnected subgraphs. A change of sign on all edges intersecting the cut set cannot alter the signature of the cycles of the network (cut sets intersect cycles in an even number of edges). Such operations are called switching equivalences in the signed graph literature [29], or gauge transformations in the spin glass literature [28]. If we think of a signed graph as a spin glass, then a structurally balanced graph corresponds to a so-called Mattis model [30], in which the “disorder” introduced by the negative edges is only apparent, and can be completely eliminated by a suitable gauge transformation (see [31] and [11] for an earlier formulation of a structurally balanced social network as a Mattis system). When applied to a monotone dynamical system, this transformation renders all entries of the Jacobian nonnegative, property known as Kamke condition in the literature [6]. Therefore the process of opinion forming of a two-party structurally balanced social network is always (dynamically) identical, up to the sign of the opinions, to that of a community with the same topology, but composed only by friends.

Applying a gauge transformation to one or more individuals means that those individuals change side on the two-faction bipartition: friends become adversaries and viceversa. In certain two-party political systems (Italy for example) these moves are not so uncommon. When such “conversions” happen, they are normally followed by sudden inversions of opinions on many political arguments. These phenomena are well-captured by the dynamical models we are proposing: an individual switching side perceives a strong influence to align himself with the opinions of his new friends.

**Figure 1:**
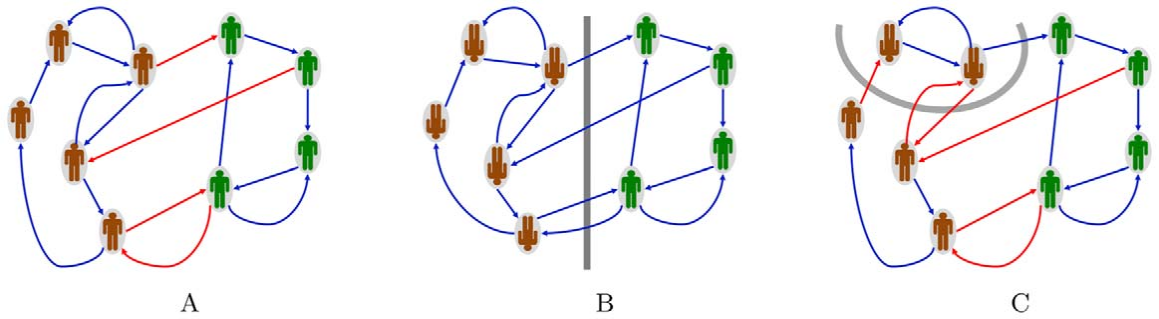
Structurally balanced community. (A): The community split into two factions such that members of the same faction are connected by friendly relationships (blue edges) and positively influence each other, while members of opposite factions are linked by adversary relationships (red edges) and negatively influence each other. All cycles and semicycles contain an even number of negative edges. (B): the gauge transformation, i.e., the switch of sign to all edges of the cut set (gray line), renders the signed graph completely blue. It corresponds to all individuals on one side of the cut set changing their mind simultaneously on their relationships with the other faction (in the drawing individuals are “flipped” for analogy with spins in Statistical Physics). (C): In this gauge transformation only the two individuals above the gray cut set switch side. The graph clearly remains bipartite. The three signed graphs in (A), (B) and (C) all are exactly structurally balanced.

## Methods

### A Dynamical Model for Influences

Consider the dynamical system
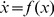
(1)where 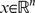 is the vector of opinions of the 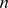 individuals and the functions 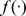 describe the process of opinion forming of the community. Assume 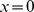 is a fixed point of (1). This is equivalent to assume that no opinion is formed unless at least one of the individuals has already an opinion at 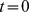, i.e., unless 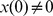 in (1).

We model the influence of the 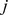-th individual over the 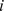-th individual by the partial derivative
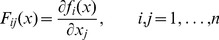
(2)so that the matrix of pairwise 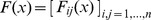 is the formal Jacobian of the system (1). We expect then that the influence of a friend is positive, 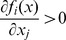, and that of an adversary negative, 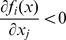. We also expect that qualitatively these influences do not change sign if we compute them in different points 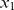 and 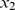 in opinions space (friends are friends in the good and in the bad moments, and so are enemies). If 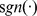 is the sign function, in formulas:



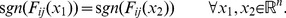
(3)The condition (3) is denoted “sign stability” in [6]. If we define 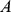 as the 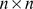 matrix of entries
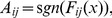
(4)at an arbitrary point 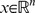, then (3) implies that 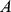 has to have the same sign pattern over the entire opinion space 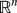.

**Figure 2:**
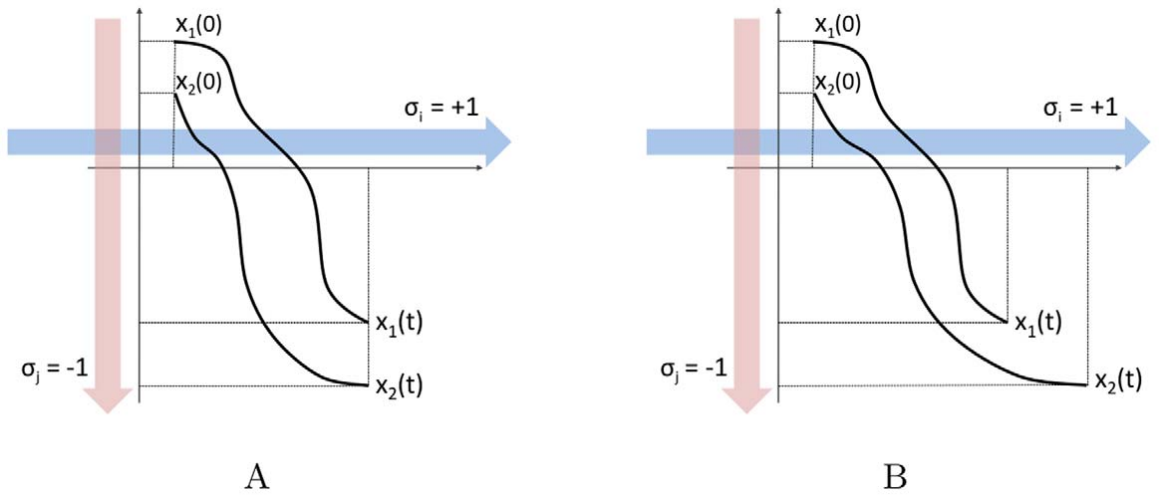
Examples of monotone and strongly monotone trajectories. Given 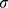, a system like (1) is monotone (panel (A)) if initial conditions 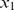, 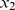 which respect the partial order 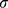 (meaning 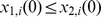 when 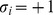, 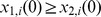 when 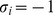) induce solutions in (1) which respect the partial order 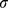 for all times (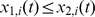 when 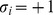, 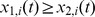 when 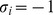). It is strongly monotone (panel (B)) if initial conditions respecting the partial order 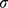 and such that they differ in at least a coordinate (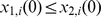 when 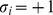, 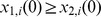 when 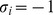, plus 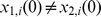 for some 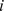) induce solutions in (1) which respect the partial order 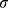 with strict inequality for all 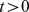 along all coordinates (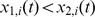 when 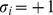, 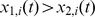 when 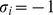).

Notice that our considerations are more general than just taking the Jacobian linearization of (1) around an equilibrium point. In particular, the system (1) may have multiple equilibrium points, even with different stability characters. This is irrelevant to our discussion. Even the precise functional form of the 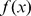 is not assumed to be known a priori, as long as it obeys (3). One possible choice for 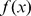 (used in the simulations of the paper) is presented in the Text S1.

Furthermore, we do not consider our own current opinion as useful to reinforce it or to change our mind. On the contrary, we will normally consider 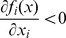, i.e., opinions are gradually forgotten over time (this helps in avoiding divergence of the ODEs (1)).

The following two different situations can be considered:

1. influences are always reciprocal:
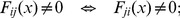
(5)
2. influences can be asymmetric

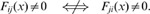
(6)


We assume henceforth that 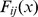 and 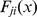 never have opposite signs:
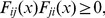
(7)condition which is called sign symmetry in [6] and which corresponds to two individuals never perceiving reciprocal influences of opposite signs. From (4), conditions analogous to (5)–(6) hold for 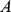: in the first case A is symmetric; in the second it need not be. The condition (7) instead becomes:
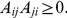
(8)


### The Associated Signed Social Community and its Structural Balance

Under the sign stability condition (3), the sociomatrix of the signed social network can be identified with the matrix 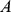. Associating social relationships with influences, as assumed here, means that the 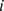-th individual considers the 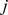-th individual a friend when 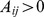, an adversary when 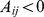, while when 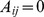 no relationship is perceived by the 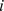-th individual. Therefore in this work the matrix 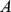 plays the double role of signature of the Jacobian of the influences and of sociomatrix of the signed social network.

For a symmetric 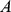, assuming that the social community is structurally balanced means that all cycles in the (undirected) graph of adjacency matrix 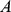 have to have positive sign [4]. When instead influences can be asymmetric, 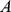 is the adjacency matrix of a digraph. In social network theory, the notion of structural balance is extended to digraphs by looking at “semipaths” and “semicycles”, i.e., undirected paths and undirected cycles of the underlying undirected signed graph obtained ignoring the direction of the edges [4], see Fig. 1(A) for an example. A necessary and sufficient condition for a digraph to admit an underlying undirected graph is (8), i.e., no negative directed cycle of length 2 exist in the signed digraph. Under this assumption, no cancellation appears when we take the “mirror” of 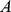 (i.e., 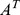) and consider 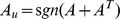 as adjacency matrix of the underlying undirected graph. When this is possible, then a directed signed network is structurally balanced if and only if all undirected cycles of 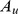 have positive sign [4].

A sociomatrix 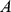 is reducible if there exists a permutation matrix 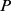 such that 
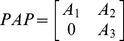
 with 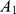, 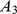 square submatrices. A is irreducible otherwise. In terms of the graph of 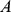, irreducibility corresponds to a strongly connected graph, i.e., a graph for which there exists a directed path between any pair of nodes (see Fig. 1 for an example of strongly connected graph and Fig. S3(A) for one of non-strongly connected graph). Irreducibility of 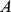 implies therefore that each individual is directly or indirectly influenced by the opinion of any of the other members of the community.

**Figure 3:**
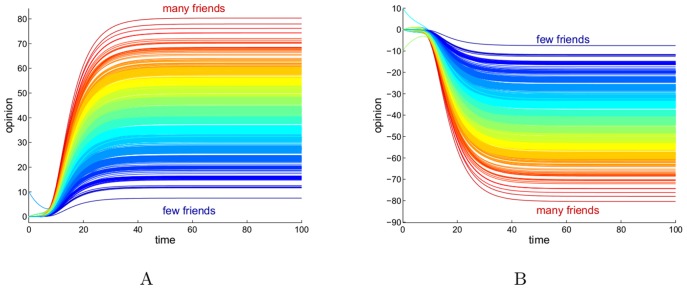
Collective opinions triggered by the opinion of one or two individuals, in the cooperative behavior case. The color of a curve is proportional to the in-degree of the individual. Individuals with the highest in-degree form the strongest opinions. In panel (A) a single 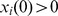 steers the whole community to a positive opinion; in panel (B) two individuals have contrasting initial conditions. When all influences are equal, the whole community is steered towards the opinion of the most connected non-zero opinioner.

### Monotone Dynamical Systems

For a thorough introduction to the theory of monotone systems, the reader is referred to [5]–[7]. In 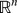, consider one of the orthants: 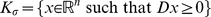 where 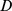 is a diagonal matrix 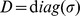 of diagonal elements 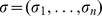, 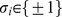, and denote by 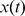 the solution of (1) at time 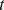 in correspondence of the initial condition 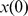. The vector 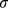 identifies a partial order for the 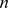 axes of 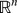, which can be the “natural” one when 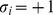, or the opposite when 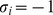, see Fig. 2.

The partial order generated by 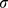 is normally indicated by the symbol “
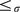
”: 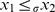 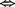 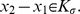 The system (1) is said *monotone* with respect to the partial order 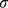 if for all initial conditions 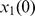, 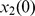 such that 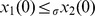 one has 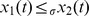 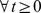. Strict ordering is denoted 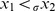 and corresponds to 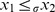, 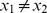, meaning that strict inequality must hold for at least one of the coordinates of 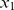, 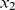, but not necessarily for all. When inequality must hold for all coordinates of 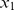, 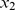 then we use the notation “
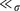
”. The system (1) is said *strongly monotone* with respect to the partial order 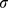 if for all initial conditions 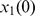, 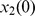 such that 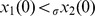 one has 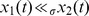 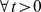. See Fig. 2 for a graphical description of these definitions.

Monotonicity of a system can be verified in terms of the Jacobian matrix 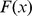, via the so-called Kamke condition ([6], Lemma 2.1), which says that the system (1) is monotone with respect to the order 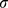 if and only if
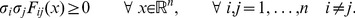
(9)


From (3)–(4), it follows that the condition (9) can be stated equivalently in terms of 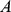 as
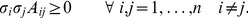
(10)


The condition (10) admits a graph-theoretical reformulation which is identical to that for structural balance (see e.g. [6]). The system (1) is monotone with respect to some orthant order if and only if all semicycles of length 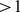 of the signed digraph of the sociomatrix 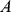 have positive sign. Therefore, under the assumption that our opinion is positively influenced by our friends and negatively by our adversaries, we can conclude that the dynamics of opinion forming in structurally balanced communities have indeed to obey a monotone dynamics.

Under the assumption (8), the condition (10) (and, similarly, (9)) covers both cases of symmetric and asymmetric influences. In fact, the non-strict inequality in (10) accounts exactly for situations in which 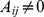 while 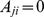, encountered in directed graphs.

If in addition to being monotone the sociomatrix 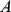 is also irreducible, then the system (1) is also strongly monotone [5]. As depicted in Fig. 2, strong monotonicity implies that opinions are strictly ordered for all individuals. In terms of our social community, this irreducibility corresponds to the fact that all individuals have some influence power over the community, even the less influential members, and strict ordering translates into the fact that no individual can remain neutral to the influences of the community. Hence, whenever an opinion is seeded all individuals have to eventually take side. See Fig. S3 for an example of monotone but not strongly monotone network. Following [7], a graph-theoretical test of strong monotonicity is that all directed cycles of the (strongly connected) digraph of 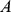 have to have positive sign.

**Figure 4:**
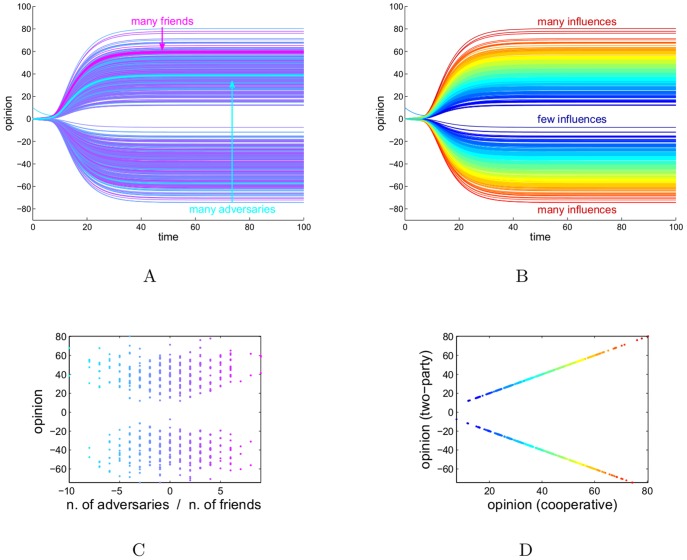
Collective opinions triggered by the opinion of a single individual, in the structurally balanced case. Both (A) and (B) show how the community becomes polarized into two factions with opposite opinions. (A): The color of a curve is proportional to the difference in the in-degree between friends and adversaries. The two highlighted curves represent the individuals with the most of friends (pink) and adversaries (cyan). (B): for the same dynamics as in (A) the color now represents the total in-degree of an individual, regardless of the sign of the relationship. Clearly the strength of an opinion depends on the total number of relationships, rather than on the proportion friends/adversaries. (C): using the color-code of (A), the scatter plot shows the steady state 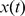 at 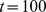 against the row sum of the sociomatrix 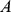 (negative sum means the individual has more adversaries than friends, positive sum the opposite). (D): using the color-code of (B), the steady state 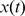 at 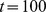 is compared with state 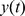 for the cooperative system one obtains gauge transforming the system with 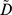 (see also Fig. S5).

## Results

### Propagation of Opinions on a Cooperative System

A particular (trivial) case of structural balance is given by 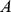 with all non-negative entries. All individuals are friends and no tension ever emerges in decision making, except perhaps for a transient evolution (due to conflicting initial conditions). The corresponding system (1) is called *cooperative* in this case [6].

We analyze the following situations for the initial conditions:

- a single individual has an opinion at 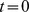;
- two different individuals have opposite opinions at 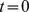.

From the definition of monotonicity, it follows that any initial condition 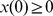, 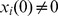 for at least one 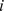, implies that 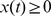 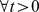. In particular, under the strong connectivity assumption, 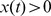 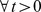, meaning that the opinion of the whole community gets influenced even by a single 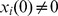. This situation is shown in Fig. 3(A) for the functional form 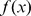 described in the Text S1 and in Fig. S1. It can be observed that the strongest opinions are achieved by the most connected individuals (red lines mean high in-degree). In a similar way, 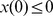 implies 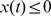 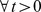 (or 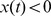 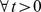 when strongly connected). The two cases represent therefore the same situation: in a cooperative system strong connectivity implies that the whole community must align itself with the opinion of the “seeder” 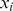. Hence in these systems seeding an idea first gives a competitive advantage over the rest of the community.

The only case in which contrast can arise in a cooperative system is when two individuals have opposite opinions at 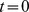. Such a contrast is not tolerated by a cooperative system, and in fact the whole community is steered to a unanimous opinion after a transient, see Fig. 3(B) for an example. Assume the 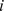-th and the 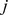-th nodes have opposite nonzero initial opinions, e.g. 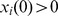 and 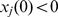. Which of these opposite initial opinions will prevail depends on the strengths of 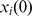 and 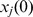, on the form of the 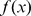 and on the connectivity of the 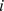-th and 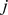-th individuals. When 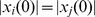 and, as in the special model described in the Text S1, all influences 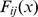 are equal in modulus, then the most out-connected individual prevails.

**Figure 5:**
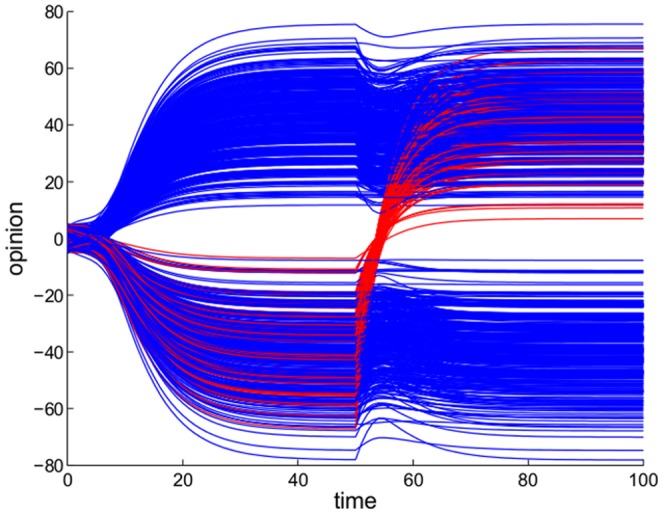
Switching party. A fraction of one of the two parties (individuals in red) joints the other faction at 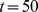. In our model this corresponds to applying a gauge transformation to the individuals in question: in the sociomatrix friends become adversaries and viceversa. This results in a change of sign of the corresponding rows/columns of the Jacobian and induces a rapid realignment of the individuals to opposite opinions. The network rapidly reaches a new steady state, differing for the old one only for the sign of the opinions of the red individuals.

### Two-party Behavior and Gauge Transformations

A well-known property of a structurally balanced signed social network is that it can be partitioned into two disjoint antagonistic subcommunities. Each community contains only friends, while any two (related) individuals from different communities are adversaries. This means that only 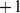 edges of 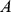 link members of the same party, while only 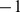 edges link members of different parties, see Fig. 1. From the sign stability condition (3), the same is true replacing 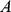 with the formal Jacobian 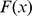. Consider the change of coordinates 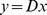, 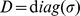 and 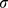 a partial ordering of 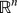. Since 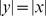 and 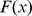 is sign constant for all 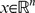, it follows that 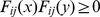. From 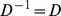, the change of variable 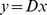 yields the new Jacobian 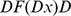. For analogy with the theory of Ising spin glasses [28], operations like
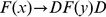
(11)are here called *gauge transformations*, and correspond to rearrangings of the order of the 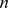 axes of 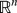 which modify the sign of the entries of the Jacobian, without altering its absolute values. In terms of the graph of 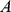, a gauge transformation 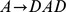 corresponds to changing sign to all edges adjacent to the nodes corresponding to the 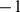 entries of 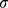. As directed cycles and semicycles share two (or zero) edges with each node, gauge transformations do not alter the signature of the cycles of the network. This is well-known in the Ising spin glass literature, see e.g. [32] (the extension to digraphs is completely straightforward). One says that operations like (11) can alter the “apparent disorder”, while the “true disorder” (or “frustration”) of the system is an invariant of (11). In particular, when 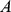 is structurally balanced the true disorder is zero. In Statistical Physics this case is called Mattis spin model [28]: an Ising model in “disguise” (the disguise being a gauge transformation). The Kamke condition rephrases this property in terms of 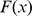. In fact, (9) implies that there exists a special ordering 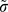 for which the gauge transformed system 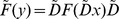, 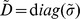, is such that 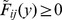 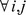, like in a cooperative system. In terms of the dynamics (1), this means that in a structurally balanced network the presence of adversaries does not alter the monotonic character of the opinion forming process: the dynamics is monotone regardless of the amount of apparent disorder present in the system. In particular, in the strongly connected case the role of the initial conditions in steering the opinion of the whole community is similar to the one described for cooperative systems. The only difference in the integral curves of (1) with respect to the cooperative case is that now the two parties converge (equally orderly) to opposite decisions, according to the faction to which each individual belongs to.

For opinions that converge to a steady state such as those represented here, the property 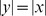 across all the gauge transformations implies that all the corresponding dynamical systems have the same convergence speed, see Fig. 4(D). We deduce therefore that in a structurally balanced community formed by friends and adversaries, the process of opinion forming is (dynamically) identical, up a to a gauge transformation, to the one described by the all-friends community. The splitting into friends and adversaries only changes the sign of the opinion vector 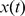 which reflects now the polarized subdivision. The case of opinions triggered by a single individual is shown in Fig. 4. In particular in this model (with the assumption of all identical kinetics adopted in the simulations, see Text S1), the strength of the opinion of an individual at steady state is not a function of the in-degree of friends or of adversaries alone, but only of the total in-degree of relationships of an individual, regardless of their sign, see Fig. 4(A) *vs* (B).

## Discussion

While the connection between structural balance and monotonicity is not new [7], the novelty of this paper is the use of this connection to draw conclusions on plausible opinion dynamics taking place on structurally balanced communities. A structurally balanced network represents a perfectly polarized community in which the drawing of a line separating friends from enemies is always an unambiguous process (compare Fig. 1 and Fig. S2). It is this lack of ambiguity that yields the high predictability of opinions. The key assumption for this to happen, that the opinions of friends exercise a positive influence and those of enemies a negative one, is realistic in this context. Most importantly, this assumption is needed only in *qualitative* terms, in the sense that it is not related to the specific values assumed by the 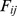 but only to their sign. This is important in our case, as the functional form of a dynamical process of opinion forming is necessarily known only in qualitative terms. The observation also implies that our results are robust, as they hold for any 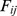 (and hence for any 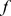) taking values in the correct orthant. For example, the reasonable scenario that an individual is much more sensitive to the opinions of his friends than to those of his enemies (i.e., that 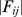 large only when 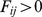) is compatible with our model. As already mentioned, in the Ising spin models of Statistical Physics, balance corresponds to lack of true disorder (or frustration, as it is commonly called in that literature). It is worth remarking that even in this context only the signs of the edges (i.e., the signs of the entries of 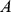) matter, rather than their specific magnitude.

In the original formulation of Heider [3], only the structural balance of triads of individuals was considered. Clearly the Cartwright-Harary generalization used here, that structural balance is equivalent to all semicycles of length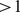 being positive [4], subsumes Heider’s length-3 cycles situations. For graphs that are not fully connected [10], [33], the opposite is not necessarily true, see Fig. S4.

A case frequently studied in the Statistical Physics literature deals with defining suitable dynamics of edge sign changes able to “steer” a frustrated network towards a structurally balanced sign configuration [10], [26], [27], [33]. This type of operations are gauge inequivalent and at each step they alter the true disorder (and hence the level of balance) of the network. Although the task is often to iteratively reduce the disorder of a given signed network and hence to obtain asymptotically a structurally balanced network, this type of process is fundamentally different from the monotonicity-induced dynamical properties of interest here, which require a “quenched”, frustration-free sociomatrix to start with.

For social communities such as those described in this work, characterized by a fixed topology (represented by the sociomatrix 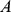) and “quenched” sign assignments to the edges, continuous-time dynamics of opinion forming is in our knowledge studied only in the non-negative sociomatrix case [34], [35]. This corresponds to a special case of what is studied here, namely the cooperative systems in which negative influences are banned. In the context of opinion forming, restricting to non-negative influences can represent an undesirable (and unnecessary) limitation. Notice how all results obtained for non-negative sociomatrices can be readily extended to signed sociomatrices by means of gauge transformations.

While many real social networks are evolving, in the sense that new links are added or removed at all times, or edge signs are switched, there are however cases where our assumptions (fixed topology, fixed signs and structural balance) are reasonable, like two-party political assemblies. Looking for example at the recent records of the US congress, a situation that describes well the significance of negative influences is the Summer 2011 deadlock on raising the national debt ceiling: in this case the antagonism and ideological divide between the two main political factions was the main origin of the legislative gridlock. Needless to say, countless similar examples can be found in basically all two-party democratic systems.

In the polarized scenario of a structurally balanced social community, a gauge transformation has also the interpretation of individuals leaving a party to join the opposite party. When this happens, friends become adversaries and viceversa (i.e., for the 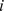-th individual the influences 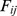 change sign). In our models, this triggers a rapid transient in which the individuals adopt the views of their new friends. An example of the realignment of opinions that follows such a move is shown in Fig. 5. Also this “turncoat”-like behavior may sound familiar in some highly polarized political contexts.

### Conclusion

In conclusion, in structurally balanced signed networks the process of opinion forming is highly predictable with no other detail than the sociomatrix. This observation suggests that the continuous-time evolution that describes the formation of the opinions in the members of the community must be itself “dynamically trivial”, although governed by ODEs which can be (plausibly) nonlinear, time-varying, coupled and high-dimensional. A number of reasons and formal analogies suggests that the class of monotone dynamical systems is a natural candidate for this role.

## Supporting Information

Figure S1
**The functional forms used for the dynamical system.** The derivative of 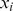 is composed of a sum of modified Michaelis-Menten functionals which are monotone in 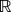 and have positive slope for a friendly relationship (blue curve in the left panel), negative slope for an adversary (red curve in the right panel). A summation of such positive/negative MM-like terms is completed by a first order degradation term (green, in both panels) which represents a forgetting factor in each individual.(TIF)

Figure S2
**Non-monotone system.** (A): Example of non-monotone (or non-structurally balanced) network. There is no bipartition of the graph such that the corresponding cut set is composed of all and only red edges. Negative cycles (and semicycles) are present in the signed graph. (B): A simulation of the dynamical system of (A) using the functional form 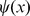 of eq. (S4) of the Text S1 yields sustained oscillations, a behavior which is unfeasible for a monotone system.(TIF)

Figure S3
**Monotone but not strongly monotone system.** (A): The graph of the network is not irreducible. The leftmost individual (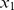 in the simulations) cannot be influenced by any of the other individuals. The dynamics of this network cannot be strongly monotone, although all directed cycles (and semicycles) are positive. (B): choosing 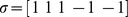, where the last two individuals are drawn in green in (A), 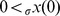 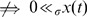, hence the system is not strongly monotone. The system is however monotone: 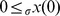 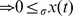. (C): 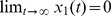 regardless of the initial condition, meaning that the individual 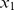 is not taking side in the decision process.(TIF)

Figure S4
**Non structurally balanced signed graph with all positive length-3 cycles.** The graph shows a social network in which all cycles of length 3 are positive, but there are cycles of length 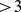 that are negative. The social network is therefore not structurally balanced, although it passes Heider test on all triads.(TIF)

Figure S5
**Two-party vs cooperative dynamics: time course.** The solution 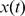 of a two-party monotone system (vertical axis) at various times is compared with its gauge-transformed cooperative system 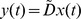 (horizontal axis). Clearly 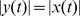 also during the transient, meaning that the convergence rate is the same across gauge equivalent systems. The color of each point is proportional to the number of relationships (friends plus enemies).(TIF)

Text S1 **A more detailed model formulation: decentralized additive nonlinear systems.**
(PDF)
