# Supplementary figures and images for: Dynamics of Opinion Forming in Structurally Balanced Social Networks
Source: PLoS One. 2012 Jun 19;7(6):e38135. doi: 10.1371/journal.pone.0038135 (PMC3378561; doi:10.1371/journal.pone.0038135)

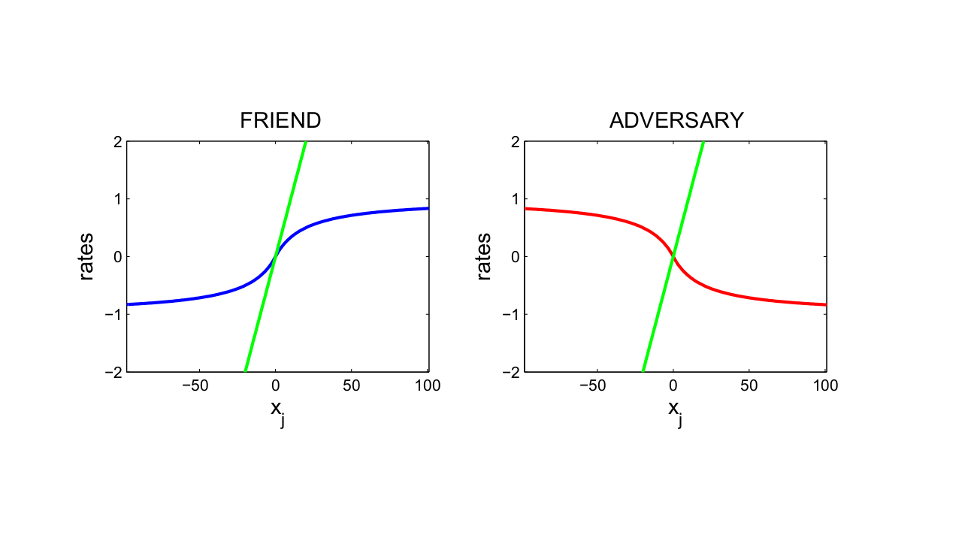

Supplement: Figure S1 — The functional forms used for the dynamical system. The derivative of is composed of a sum of modified Michaelis-Menten functionals which are monotone in and have positive slope for a friendly relationship (blue curve in the left panel), negative slope for an adversary (red curve in the right panel). A summation of such positive/negative MM-like terms is completed by a first order degradation term (green, in both panels) which represents a forgetting factor in each individual. (TIF) [file pone.0038135.s001.tif]

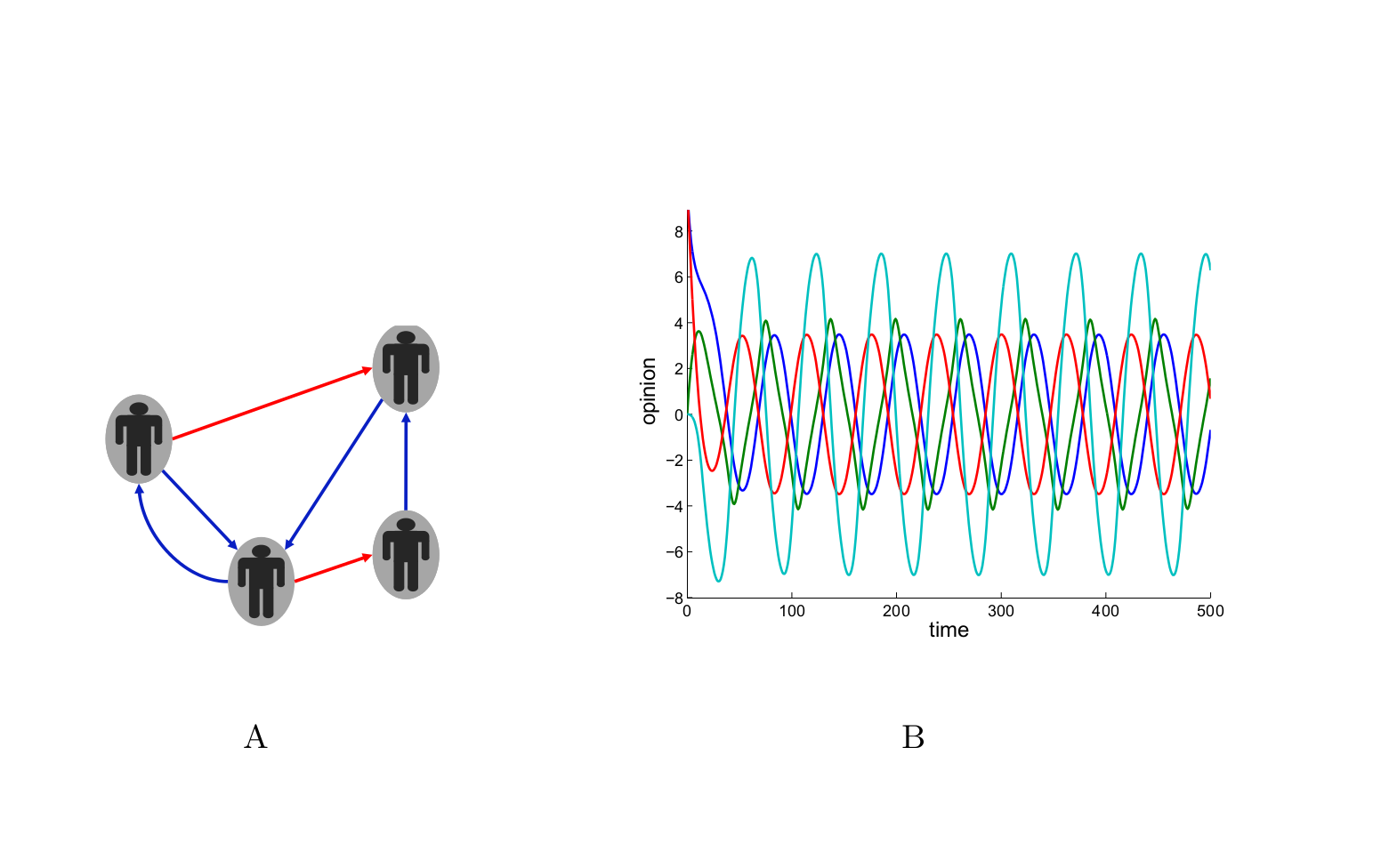

Supplement: Figure S2 — Non-monotone system. (A): Example of non-monotone (or non-structurally balanced) network. There is no bipartition of the graph such that the corresponding cut set is composed of all and only red edges. Negative cycles (and semicycles) are present in the signed graph. (B): A simulation of the dynamical system of (A) using the functional form of eq. (S4) of the Text S1 yields sustained oscillations, a behavior which is unfeasible for a monotone system. (TIF) [file pone.0038135.s002.tif]

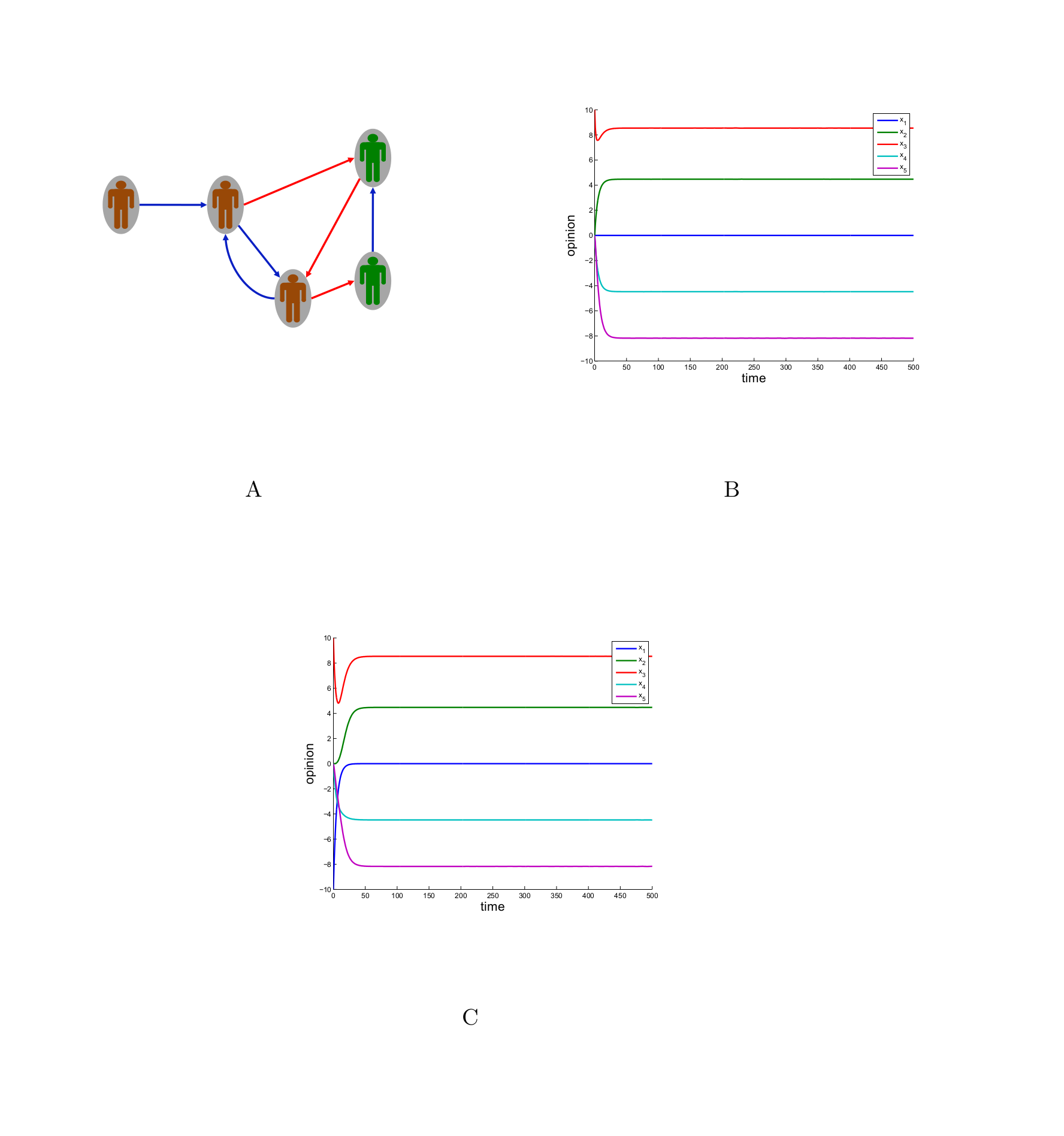

Supplement: Figure S3 — Monotone but not strongly monotone system. (A): The graph of the network is not irreducible. The leftmost individual ( in the simulations) cannot be influenced by any of the other individuals. The dynamics of this network cannot be strongly monotone, although all directed cycles (and semicycles) are positive. (B): choosing , where the last two individuals are drawn in green in (A), , hence the system is not strongly monotone. The system is however monotone: . (C): regardless of the initial condition, meaning that the individual is not taking side in the decision process. (TIF) [file pone.0038135.s003.tif]

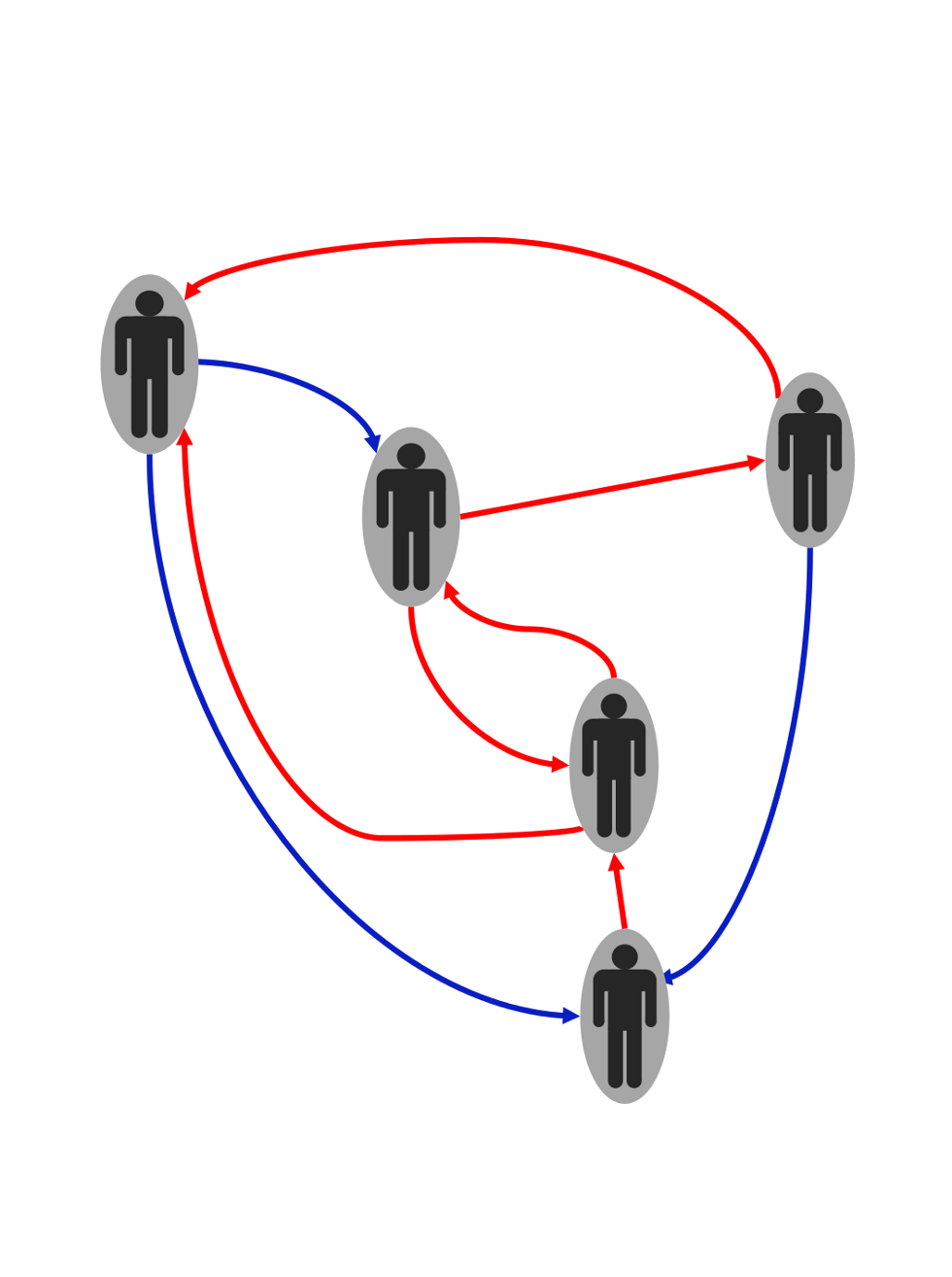

Supplement: Figure S4 — Non structurally balanced signed graph with all positive length-3 cycles. The graph shows a social network in which all cycles of length 3 are positive, but there are cycles of length that are negative. The social network is therefore not structurally balanced, although it passes Heider test on all triads. (TIF) [file pone.0038135.s004.tif]

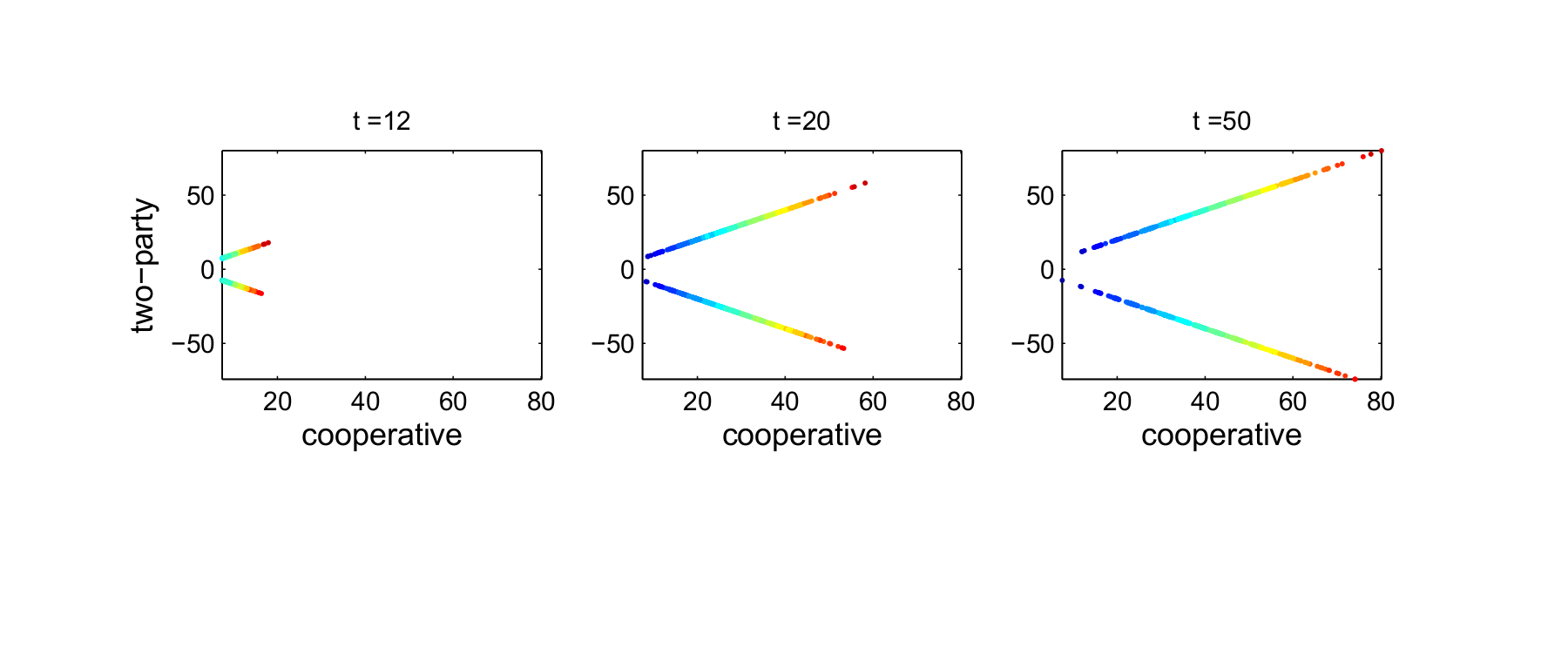

Supplement: Figure S5 — Two-party vs cooperative dynamics: time course. The solution of a two-party monotone system (vertical axis) at various times is compared with its gauge-transformed cooperative system (horizontal axis). Clearly also during the transient, meaning that the convergence rate is the same across gauge equivalent systems. The color of each point is proportional to the number of relationships (friends plus enemies). (TIF) [file pone.0038135.s005.tif]
