# Supplementary material for: Dynamics of Opinion Forming in Structurally Balanced Social Networks
Source: PLoS One. 2012 Jun 19;7(6):e38135. doi: 10.1371/journal.pone.0038135 (PMC3378561; doi:10.1371/journal.pone.0038135)
Supplement: Text S1 — A more detailed model formulation: decentralized additive nonlinear systems. (PDF) [file pone.0038135.s006.pdf]

SUPPLEMENTARY TEXT: Dynamics of opinion forming in structurally balanced social networks

Claudio Altafini,
SISSA, International School for Advanced Studies,
Trieste, Italy
E-mail: altafini@sissa.it

A more detailed model formulation: decentralized additive nonlinear systems

As mentioned in the paper, any sufficiently regular $f(\cdot) : \mathbb{R}^n \to \mathbb{R}^n$ whose formal Jacobian $F(x)$ obeys to the Kamke condition is acceptable for the purposes of this study. In this Section we introduce a special form for the ODEs (1) of the paper, which is then used to simulate monotone dynamical systems. This special form fits naturally in our problem setting and it is general enough to capture all the features of interest of monotone dynamical systems.

For the i-th component of the vector field $f(x)$ in (1) consider the following ODE:

$$\dot{x}_i = f_i(x) = \sum_{j=1}^{n} A_{ij}\psi_{ij}(x_j) - \lambda_i x_i, \tag{S1}$$

$i = 1, \ldots, n$, where

- $\psi_{ij} = \psi_{ij}(x_j)$ is Lipschitz continuous, depends only on the j-th state and is such that $\frac{\partial \psi_{ij}}{\partial x_j} \geqslant 0$, $\forall\, x_j \in \mathbb{R}$;
- $\lambda_i > 0$, i.e., an exponential decay affects the opinions of all individuals;
- $A_{ij} \in \{0, \pm 1\}$ and $A = (A_{ij})$ structurally balanced, that is, $\exists\, \tilde{D} = \text{diag}(\tilde{\sigma})$ such that $\tilde{D}A\tilde{D}$ has all nonnegative entries.

The form (S1) corresponds to an opinion forming process in which at each node the influences of the neighbors is additive, weighted by the signed sociomatrix A, and damped by the forgetting factor λ_i. The functionals $\psi_{ij} : \mathbb{R} \to \mathbb{R}$ are local (i.e., only the state x_j of the j-th individual matters) and nondecreasing: the higher is x_j the higher $\psi_{ij}(x_j)$. The monotonicity of the functionals reflects the fact that the opinion expressed by the j-th individual (i.e., $\psi_{ij}(x_j)$) in his/her relationships is coherent with his/her "true" opinion (i.e., x_j). The sociomatrix A specifies if the opinion expressed by the j-th individual ($\psi_{ij}(x_j)$) influences positively or negatively the i-th neighbor (i.e., if $A_{ij}\psi_{ij}(x_j)$ is positive or negative). The degradation term λ_i has the form of an exponential decay, and models a forgetting factor in the opinion of each individual. Notice that since structural balance and monotonicity are checked on the off-diagonal part of the Jacobian, the presence of λ_i does not interfere with these properties of the system. It is however necessary to have the solutions of (S4) not diverging to $\pm\infty$. Eq. (S1) can therefore be written as

$$f_i(x) = \sum_{j \in friends(i)} \psi_{ij}(x_j) - \sum_{j \in advers(i)} \psi_{ij}(x_j) - \lambda_i x_i. \tag{S2}$$

A special case of (S2) is the following

$$\psi_{ij}(x_j) = \psi_j(x_j) \qquad \forall\, i = 1, \ldots, n$$

corresponding to a node influencing all its neighbors in the same manner (up to the sign, which is contained in A). In this case, (1) of the paper can be written more compactly as

$$\dot{x} = A\psi(x) - \Lambda x \tag{S3}$$

where $\psi(x) = \begin{bmatrix}\psi_1(x_1) & \dots & \psi_n(x_n)\end{bmatrix}^T$ and Λ is a diagonal matrix $\Lambda = \text{diag}(\begin{bmatrix}\lambda_1 & \dots & \lambda_n\end{bmatrix})$. Nonlinear systems with a structure like (S3) are sometimes called Persidskii systems [1].

In this study we have chosen to work with monotonic functionals derived by the so-called Michaelis-Menten kinetic forms, widely used in mathematical biology to describe reaction rates [2]. These functions are the following:

$$\psi_j(x_j) = \frac{x_j}{\theta_j + |x_j|}, \qquad x_j \in \mathbb{R} \tag{S4}$$

where $\theta_j > 0$ is called the half-rate constant, and represents the value of x_j at which ψ_j reaches the value of 1/2. The peculiarity of the ψ_j is in fact that the rate it describes saturates for large values of x_j:

$$lim_{x_j \to \pm\infty} \psi_j(x_j) = \pm 1,$$

i.e., opinions are not formed too quickly. The absolute value in the denominator of (S4) is needed to have a well-defined expression also for negative x_j (in biological models x normally represents concentrations, hence the negative values are disregarded). The functional form of ψ_j is shown in Fig. S1. Since

$$\frac{\partial \psi_j(x_j)}{\partial x_j} = \frac{\theta_j}{(\theta_j + |x_j|)^2} > 0$$

from (S2) one has

$$F_{ij}(x) = \frac{\partial f_i(x)}{\partial x_j} = \begin{cases} \frac{\theta_j}{(\theta_j + |x_j|)^2} & \text{if } j \in friends(i) \\ \frac{-\theta_j}{(\theta_j + |x_j|)^2} & \text{if } j \in avers(i) \end{cases} \tag{S5}$$

i.e., $\text{sgn}(F_{ij}(x))$ is always constant for all $x \in \mathbb{R}^n$, as required for monotone systems. In particular, then, a change of partial order σ on the axes, $y = Dx$, $D = \text{diag}(\sigma)$, does not alter the Jacobian $F(x)$. From (S5), in fact, $F_{ij}(x) = F_{ij}(y)$. From the sign stability of $F(x)$, it follows that $\text{sgn}(F_{ij}(x)x_j) = \text{sgn}(A_{ij}\psi_{ij}(x_j))$, which is enough to guarantee that the Jacobian linearization faithfully reflects the behavior of the original nonlinear system.

In the simulations of the paper, the half-rates θ_j and the degradation rates λ_i are fixed all equal.

References

1. Kaszkurewicz E, Bhaya A (2000) Matrix diagonal stability in systems and computation. Boston: Birkhäuser.
2. Edelstein-Keshet L (2005) Mathematical Models in Biology. SIAM Classics in Applied Mathematics.
